# 

**Published:** 1894-01

**Authors:** 


					﻿Vol Xiv.
JANUARY, 1894.
NO. 1.
THE
HOMEOPATHIC pHY^lClAJl,
A Monthly Journal of Homoeopathic Materia Medica
and Clinical Medicine.
•* He is a freeman whom truth makes free, and all are slaves beside."
"Seek the Truth: come whence it may, cost what it will."
EDITED AND PUBLISHED BY
WALTER M. JAMES, M. D.
PHILADELPHIA :
No. 1125 SPRUCE STREET.
. 3u O XT 3d O.3T '
ALFRED HEATH & CO., Sole Agents for Great Britain,
114 Ebury Street, Eaton Square.
-Yearly Subscription, $2.SO, invariably in advance. Single numbers, 2S etc.
Entered at the Philadelphia Peat-Offioe aa SeooodClaaa Mall Matter.
				

